# Epistaxis

**Published:** 1878-01-20

**Authors:** B. W. Barton


### *EPISTAXIS.*

To stop bleeding from the nose, it is not always enough to blow styptic or astringent powders into the nostrils; and the injection of stronger agents, while it may stop the flow of blood, is often attended with very objectionable accidents. I believe that I once endangered the life of a patient by the injection of Monsel's solution into his nostrils. Some of the solution flowed back into the larynx and trachea, and produced most painful symptoms at the moment and was followed by a degree of œdema of these parts which proved unpleasantly serious. The injection may have been done in a bungling manner, but even with skillful hands it is easy to see that such an accident might happen. Besides this the injection of this liquid is almost certain to give rise to quite profuse salivation, and if it pass into the stomach, to vomiting which is likely to undo all that has been done to arrest the bleeding. The last resort to which we flee when the simpler methods fail, that of sound and tampon, is certainly most efficient in stopping the hemorrhage, but is also a most troublesome operation if the patient should happen to be a peevish child. I have been told also that the presence of the tampons gives rise to peculiarly painful sensations.

In view of this heap of difficulties, I propose a simple method to which the foregoing objections can not be urged and which has proved, on three occasions, all that could be desired in checking the nose-bleed. I used the Monsel's Iron Solution, but applied it with feathers. The wing feather of a common fowl si most readily gotten. The barbed end, of course, is dipped into the solution and pushed rapidly back into the nostril, and turned once or twice in the fingers. In a few seconds the feather refuses to yield to pushing or pulling, showing that a firm clot has been formed. The projecting end is clipped so as not to inconvenience the patient, enough of it being left to be easily seized and removed when required. If one feather should fail to stop the blood, a second may be introduced in the same manner alongside of the first one. At the end of a certain time, the clots slough away from the nasal walls, and may be removed without trouble.

This is a very simple procedure, and I doubt whether it will fail when any other method would succeed.—*B. W. Barton, M.D., in Maryland Medical Journal.*

